# Prognostic factors for femoropopliteal vascular injuries: surgical decisions matter

**DOI:** 10.1590/1677-5449.202300502

**Published:** 2023-11-13

**Authors:** Adenauer Marinho de Oliveira Góes, Fernanda Beatriz Araújo de Albuquerque, Matheus Oliveira Feijó, Flávia Beatriz Araújo de Albuquerque, Luciana Roberta do Vale Corrêa, Mariseth Carvalho de Andrade

**Affiliations:** 1 Universidade Federal do Pará - UFPA, Belém, PA, Brasil.; 2 Universidade Federal do Paraná - UFPR, Hospital das Clinicas - HC, Curitiba, PR, Brasil.; 3 Força Aérea Brasileira - FAB, Belém, PA, Brasil.; 4 Exército Brasileiro - EB, Macapá, AP, Brasil.; 5 Hospital Metropolitano de Urgência e Emergência - HMUE, Ananindeua, PA, Brasil.; 6 Fundação Santa Casa de Misericórdia do Pará - FSCMPA, Belém, PA, Brasil.

**Keywords:** vascular system injuries, lower extremities, vascular surgical procedures, prognosis

## Abstract

**Background:**

Lower limbs are frequently involved in vascular trauma, but it is still not clear which factors lead to unfavorable clinical outcomes.

**Objectives:**

To determine the clinical profile of patients with femoropopliteal injuries, the trauma mechanisms, and treatment and identify which factors led to unfavorable outcomes.

**Methods:**

A retrospective study based on the medical records of patients treated from 2017 to 2021. The following data were assessed: sex, age, distance to reach the hospital, trauma mechanism, hypovolemic shock, additional injuries, treatment of vascular injuries, whether fasciotomy was needed, inappropriate intraoperative decisions, and injury severity score. Need for surgical reintervention, amputation, and death were defined as unfavorable outcomes. Univariate, bivariate, and logistic regression analyses were conducted.

**Results:**

The sample comprised 94 patients; 83% were men; mean age was 30.8 years; combined arterial and venous injuries prevailed (57.5%); and superficial femoral vessels were the most affected (61.7%). Penetrating mechanisms prevailed (80.9%). Arterial injuries were most frequently treated with venous graft (59.6%) and venous injuries underwent ligation (81.4%). In 15% of cases, inappropriate surgical decisions were detected; most often use of the ipsilateral great saphenous vein for arterial reconstruction. Unfavorable outcomes occurred in 44.7%: surgical reintervention was necessary in 21.3% and limb amputation in 25.5%, while 9.5% of the patients died.

**Conclusions:**

These injuries mainly involved young men, victims of gunshot wounds. Superficial femoral vessels were the most injured; concomitant non-vascular trauma was frequent, mainly fractures. Inappropriate surgical decisions increased the need for reinterventions by 34 times. Need for fasciotomy, presence of fracture/dislocation, blunt trauma mechanism, and popliteal artery injury increased the risk of amputation.

## INTRODUCTION

Although the victims of traumatic vascular injuries are primarily young men,^1-8^ patients may be of all ages and either sex. The frequency of these traumatisms has been growing and it is estimated that around 6% of civilian traumas involve vascular injury,^2^ while they occur in up to 17.6% of military traumas.^2-4,8-12^

Involvement of limb vessels is common and is associated with risk of death and amputation.^4,5^ Many different factors influence development of unfavorable clinical outcomes in cases of vascular traumatisms involving the limbs. One of the first factors to be established, more than 70 years ago,^13^ was ischemia duration, which is directly correlated with the probability of limb amputation.^14-19^

The site and mechanism of injury also affect prognosis.^1,20,21^ It is known that hemorrhage related to injuries to the femoral vessels can cause mortality of up to 8%^1,22^ and that injuries to the popliteal artery, where collateral circulation is less developed, are associated with amputation rates of up to 26%.^5,18,19,23,24^ There is also consensus that blunt traumas have worse prognosis than penetrating traumas.^1,18,21,25-27^

However, there are still unresolved issues: references in the literature on venous injuries are divided regarding whether venous ligation increases the risk of amputation^14,28,29^ and no studies could be found that assessed whether inappropriate surgical decisions affect patient prognosis. The objective of this study is to determine the profile of femoropopliteal vascular injury victims, the mechanisms of trauma, and the techniques employed to treat them and determine which factors influence the development of unfavorable clinical outcomes.

## METHODS

This study was approved by the Institutional Ethics Committee (CAAE 2114919.8.0000.5169, decision number 4928779). This is a retrospective analytical study based on data from electronic patient records from January 2017 to December 2021. All medical records containing the terms “femoral” or “popliteal” were selected and reviewed. Patients of both sexes aged over 16 years who had undergone surgical treatment for traumatic injuries to common femoral, superficial femoral, or popliteal veins or arteries caused by any mechanism were selected for the analysis.

Patients were excluded if they had been operated at other hospitals and then transferred for reassessment, those who underwent primary amputation, suffered a traumatic limb amputation, had potentially fatal concomitant injuries that could introduce confounding bias to the analysis of outcomes (cardiac traumas, injuries to other blood vessels, major abdominal viscera traumas, fractures of the pelvis, massive hemothorax, head and brain traumas, or other injuries that could cause early patient death), or if there were data missing from their medical records. Patient sex and age were analyzed, with the following age groups: less than 30 years old, 30 to 39, and over the age of 40 years. The distance from the location where the trauma occurred to the hospital was measured using Google Maps® and classified as less than or greater than 100 km.

The mechanisms of trauma were classified as penetrating (gunshot and knife wound) or blunt (traffic accidents, falls, and other mechanisms), and the vascular structure involved was recorded. Hypovolemic shock at admission was defined as systolic blood pressure less than 90 mmHg or heart rate greater than 100 beats per minute and the Injury Severity Score (ISS) was calculated^30^ for each trauma.^30-32^

Concomitant non-vascular injuries were classified as skeletal, thoracic, abdominal/pelvic, or head/neck injuries. Arterial and venous injuries were classified as section, thrombosis, pseudoaneurysm, or arteriovenous fistula. Treatment techniques were categorized as venous graft, prosthetic graft, end-to-end anastomosis, thrombectomy, arteriorrhaphy, venorrhaphy, patch, temporary shunt, venous ligation, or anticoagulation. Use of fasciotomy was also analyzed.

Use of the great saphenous vein ipsilateral to the injury for vascular reconstruction, primary arteriorrhaphy, and thrombectomy followed by arteriorrhaphy (without parietal debridement/resection of the damaged segment), and also failure to detect injuries during vascular exploration were classified as “inappropriate surgical decisions”. Surgical reintervention was defined as the need for another intervention by a vascular surgeon for debridement, late fasciotomy, or extension of a fasciotomy performed in the initial intervention. Need for reintervention and progression to amputation or death were defined as unfavorable outcomes and correlated with the variables described above.

Statistical analyses were conducted using Microsoft Office Excel® 2016 and BioEstat® 5.4. Analytical statistics were used to evaluate the results for categorical variables, the G and chi-square tests were used for univariate analyses, and the G test was used for bivariate comparisons. The Spearman correlation test was used for variables with significance in relation to unfavorable outcomes, and, after identification of correlated variables, logistic regression equations were used to calculate probabilities in relation to dependent variables. A significance level of α = 0.05, or 5%, was adopted.

## RESULTS

**Figure 1:**
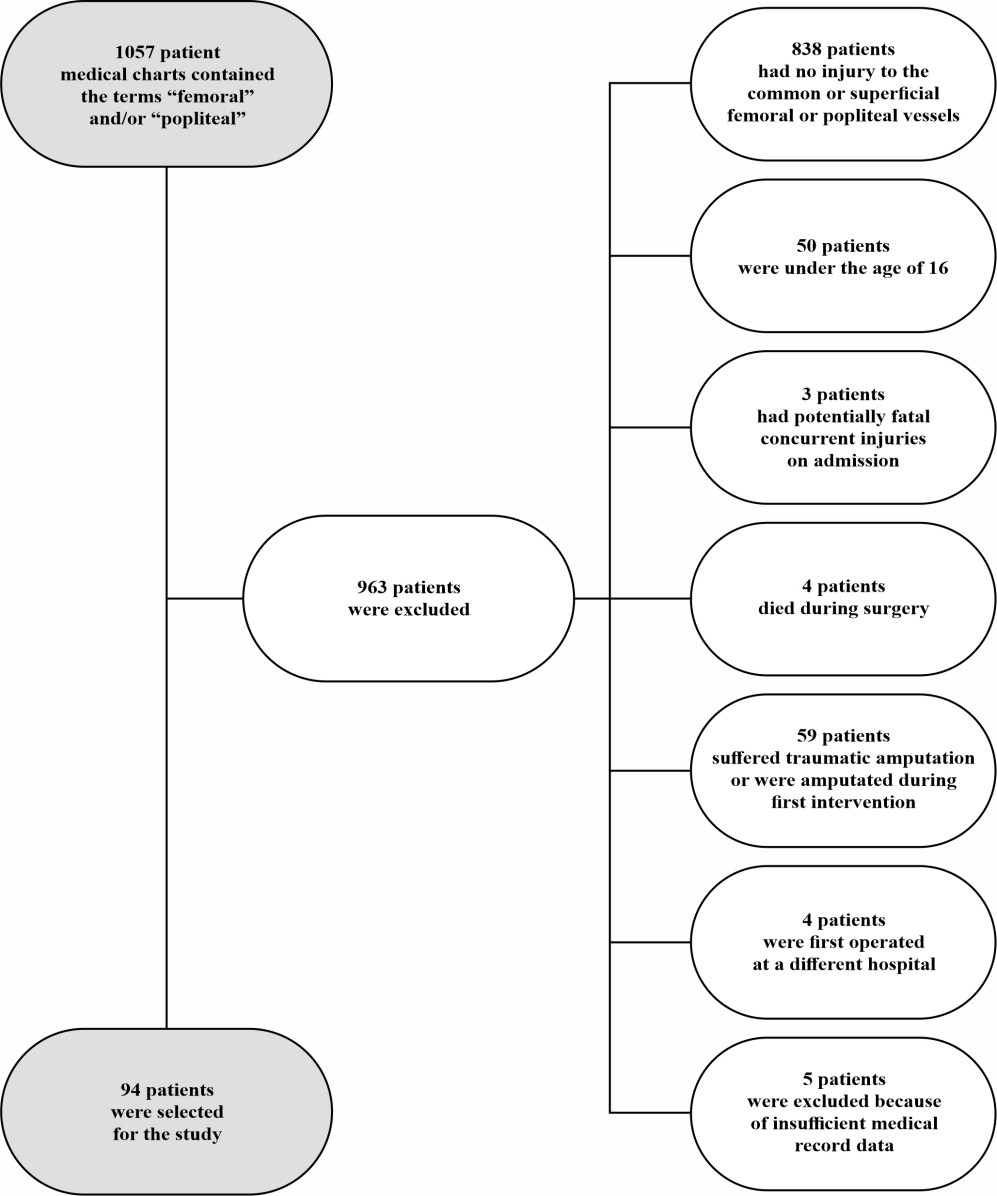
Flowchart illustrating composition of the sample.

The initial search identified 1,057 medical records. Ninety-four patients were selected after application of the inclusion and exclusion criteria. The selection process used to constitute the sample is illustrated in Figure 1. The sample comprised 78 male patients (83%) and 16 females (17%) (*p < 0.0001). Patient age ranged from 16 to 70 years, with a mean of 30.8 years, and 54.3% of the patients (51/94) were less than 30 years old (*p < 0.0001). It was possible to establish the transportation route to the point of care in 94.7% of cases (89/94), which was less than 100 km in 47.9% (45/94) of the sample and longer than 100 km for 46.8% of the patients (44/94, p = 0.9156). Additional injuries were present in 55.3% of cases (52/94) (p = 0.3023). Skeletal traumas were the most common of these, in 69.2% of the patients (*p = 0.0055) (Table 1).

**Table 1:** Sociodemographic variables and additional injuries.

**Variables**	**N**	**%**	***p*-value**
**Sex**			< 0.0001*
Female	16	17.0%	
Male*	78	83.0%	
**Age group**			< 0.0001*
< 30*	51	54.3%	
30 to 39	24	25.5%	
> = 40	19	20.2%	
**Minimum/mean/maximum**	**16 / 30.8 / 70 years**		
**Distance (km)**			0.9156
Up to 100*	45	47.9%	
More than 100	44	46.8%	
Not recorded	5	5.3%	
**Additional injuries**			0.3023
Present	52	55.3%	
Absent	42	44.7%	
**Injured structure**	**52**||	**55.3%**	**0.0055** †
Appendicular skeleton*‡	36¶	69.2%	
Other vascular injuries§	13	25.0%	
Abdomen/pelvis	9	17.3%	
Thorax	3	5.8%	
Head/neck	1	1.9%	

* Chi-square test of adherence;

† G test of adherence; N: number of cases; %: percentage of cases;

‡ Fracture: calcaneus (1), tibia (15), fibula (2), femur (20), carpal (1), radius (3), ulna (2), humerus (2); luxations were detected of the: knee (5) and hips (2);

§ other vascular injuries: deep femoral artery (4), anterior tibial artery (2), pudendal vessels (1), tibioperoneal trunk (2), external iliac vein (1), deep femoral vein (1), genicular artery (1);

|| N is the number of patients with injuries to other structures; some patients had injuries to more than one structure, in addition to the vascular injuries being studied;

¶ Some patients had injuries to more than one appendicular skeleton structure.

Combinations of arterial and venous injuries were statistically more common than either arterial or venous injuries alone (*p < 0.0001), accounting for 57.5% (54/94) of cases. The vessel most often involved was the superficial femoral (61.7%) (*p = 0.0233). One arterial injury was identified in 94.7% (89/94) (*p < 0.0001) of the sample. The artery most often involved was the superficial femoral (62.9%) (*p = 0.0197), followed by the popliteal (30.3%) and common femoral (11.2%) arteries. Venous injuries were observed in 62.8% (59/94) of cases (*p = 0.0039), with the superficial femoral vein injured in 52.5% of cases (*p = 0.0013), followed by the popliteal (40.7%) and common femoral (13.6%) veins (Table 2).

**Table 2:** Injured vascular structures.

**Variables**	**N**	**%**	***p*-value**
**Injured** v**ascular structures**			< 0.0001*
Artery and vein*	54	57.5%	
Artery only	35	37.2%	
Vein only	5	5.3%	
**Injured vessels**			
Superficial femoral vessels*	58	61.7%	0.0233*
Popliteal vessels	31	33.0%	
Common femoral vessels	14	14.9%	
Injured arteries*	**89** †	**94.7%**	< 0.0001*
Superficial femoral*	56	62.9%	0.0197*
Popliteal	27	30.3%	
Common femoral	10	11.2%	
Injured veins*	**59^†^**	**62.8%**	0.0039*
Superficial femoral*	31	52.5%	0.0013*
Popliteal	24	40.7%	
Common femoral	8	13.6%	

* Chi-square test of adherence; N: number of cases; %: percentage of cases;

† N is the number of patients who had at least one injury to one of the arteries or veins being studied; there were patients who had more than one of the arterial/venous injuries studied.

Penetrating trauma mechanisms were the most prevalent, in 80.9% (76/94) (* p < 0.0001). Of these, gunshot wounds (92.1%) were more common than knife wounds (7.9%) (*p < 0.0001). All of the blunt trauma cases were the result of traffic accidents.

The majority (52.8%) of vascular injuries, whether arterial of venous (40.2%), were classified as sectioned vessels (partial/total) (*p < 0.0001). Arterial injuries were most frequently treated with venous grafts (59.6%) or end-to-end anastomosis (23.6%), while the majority of venous injuries were treated by venous ligation (81.4%) or venorrhaphy (13.6%) (*p < 0.0001) (Table 3).

**Table 3:** Characteristics of vascular injuries and treatments employed.

**Variables**	**N**	**%**	***p*-value**
**Trauma mechanism**			< 0.0001*
Penetrating*	**76**	**80.9%**	
Gunshot	70	92.1%	
Knife wound	6	7.9%	
**Blunt**	**18**	**19.1%**	
Traffic accidents	18	100.0%	
**Description of arterial injury**	**89** †		< 0.0001*
Partial/total section*	47	52.8%	
Thrombosis	16	18.0%	
Pseudoaneurysm	7	7.9%	
Arteriovenous fistula	6	6.7%	
Not recorded	18	20.2%	
**Arterial treatment**			< 0.0001*
Venous graft*	53	59.6%	
End-to-end anastomosis	21	23.6%	
Fogarty thrombectomy	5	5.6%	
Ligation	4	4.5%	
Arteriorrhaphy	3	3.4%	
Prosthetic graft	1	1.1%	
Patch	1	1.1%	
Arterial shunt	1	1.1%	
**Description of venous injury**	**59^†^**		< 0.0001*
Partial/total section*	29	49.2%	
Arteriovenous fistula	6	10.2%	
Thrombosis	1	1.7%	
Not recorded	23	39.0%	
**Venous treatment**			< 0.0001*
Ligation*	48	81.4%	
Venorrhaphy	8	13.6%	
Anticoagulation	1	1.7%	
Not recorded	2	3.4%	

* G test of adherence; N: number of cases; %: percentage of cases;

† N is the number of patients who had at least one injury to one of the arteries or veins being studied; there were patients who had more than one of the arterial/venous injuries studied.

In the majority of cases, review of the descriptions of surgery did not reveal any inappropriate surgical decisions. Inappropriate surgical decisions were identified in 15.9% of cases (15/94) (*p < 0.0001). The most common of these was use of the great saphenous vein from the injured limb as venous graft material, observed in 10 patients. Other cases involved arteriorrhaphy and thrombectomy without resection of the injured segment and cases in which vascular exploration failed to detect injuries that were present and were diagnosed later when the patient’s clinical status deteriorated. Unfavorable outcomes occurred in 44.7% of cases (42/94) (p = 0.1891), 38.1% (16/42) comprising amputation only, 23.8% (10/42) comprising reintervention only, and 11.9% (5/42) comprising death only. In 11 cases there was more than one of these unfavorable outcomes (Table 4).

**Table 4:** Unfavorable outcomes and inappropriate surgical decisions.

**Variables**	**N**	**%**	***p*-value**
**Inappropriate surgical decisions**			**< 0.0001** *
Present	15	16.0%	
Absent*	79	84.0%	
**Unfavorable outcome**			0.1891
Yes	42	44.7%	
No	52	55.3%	
Type of unfavorable outcome*			0.5465
Amputation	24	25.5%	
Reintervention	20	21.3%	
Death	9	9,60%	
**Combinations of unfavorable outcomes**	**42**		0.0255*
Amputation only*	16	38.1%	
Reintervention only	10	23.8%	
Death only	5	11.9%	
Amputation + reintervention	7	16.7%	
Reintervention + death	3	7.1%	
Amputation + death	1	2.4%	

* G test of adherence; N: number of cases; %: percentage of cases.

**Figure 2:**
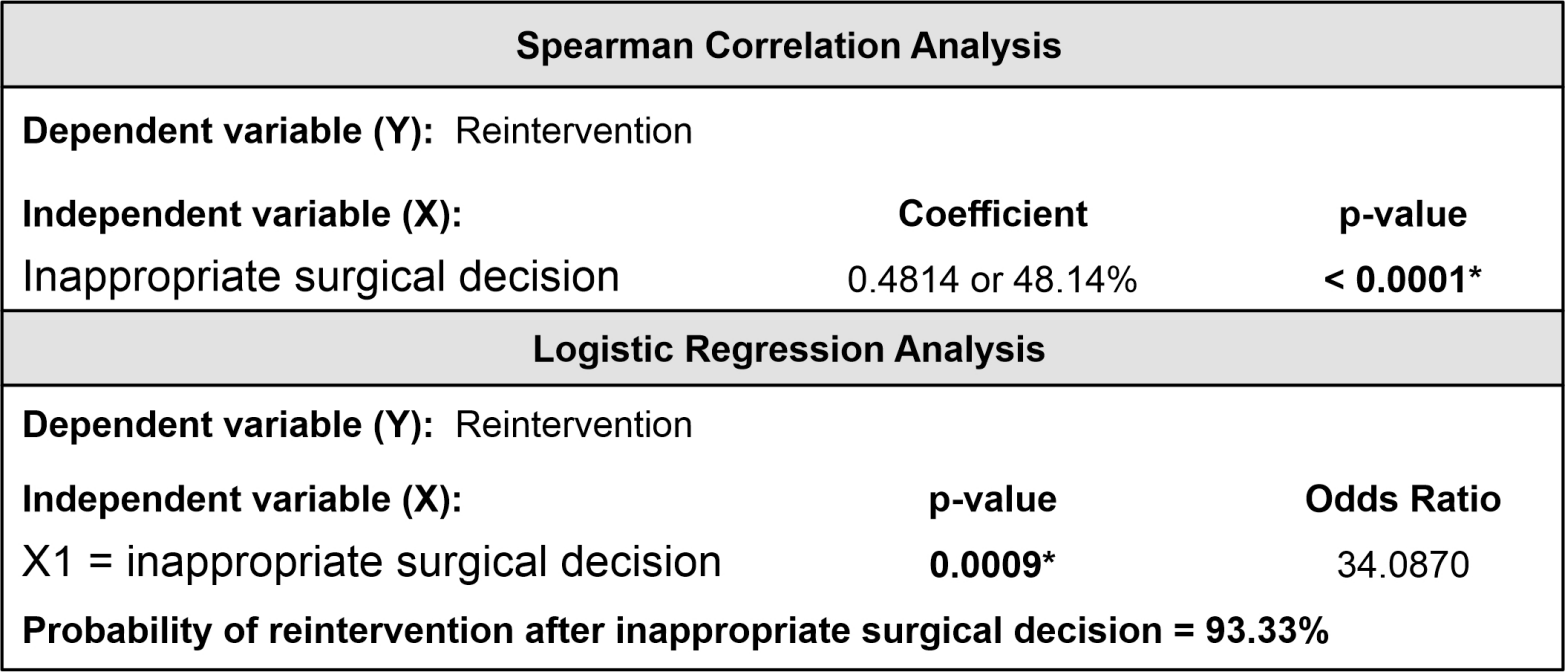
Spearman correlation analysis and logistic regression for the variable reintervention. *Spearman's correlation coefficient.

When need for reintervention was analyzed, there was a statistically significant association with inappropriate surgical decisions (*p = 0.0001). When this association was not detected, the reintervention rate was 29.1%, but when it was present the rate was 93.3% (Table 5), equating to a 34.1 times increase in the probability of surgical reintervention (Figure 2). The most frequent reinterventions were debridement (10) and fasciotomy or extension of prior fasciotomy (8), followed by venous graft (5), thrombectomy (4), arterial venous ligation (1), and pseudoaneurysm repair(1).

**Table 5:** Unfavorable outcome reintervention and relationships with the other study variables.

**Variables**	**N**	**Surgical reintervention**				
		**Yes (n=20)**		**No (n=74)**		***p*-value**
**Superficial femoral vessels (n=58)**						0.1681
Artery and vein	**32**	8	25.0%	24	75.0%	
Artery only	**24**	2	8.3%	22	91.7%	
Vein only	**2**	0	0.0%	2	100.0%	
**Popliteal vessels (n=31)**						0.2593
Artery and vein	**21**	8	38.1%	13	61.9%	
Artery only	**7**	2	28.6%	5	71.4%	
Vein only	**3**	0	0.0%	3	100.0%	
**Common femoral vessels (n=14)**						0.4069
Artery and vein	**6**	1	16.7%	5	83.3%	
Artery only	**6**	0	0.0%	6	100.0%	
Vein only	**2**	0	0.0%	2	100.0%	
**Needed fasciotomy**						0.1571
Yes	**30**	9	30.0%	21	70.0%	
No	**64**	11	17.2%	53	82.8%	
**Fracture and/or luxation**						0.3543
Yes	**34**	9	26.5%	25	73.5%	
No	**60**	11	18.3%	49	81.7%	
**Trauma mechanism**						0.9554
Gunshot	**70**	15	21.4%	55	78.6%	
Traffic accident	**18**	4	22.2%	14	77.8%	
Knife wound	**6**	1	16.7%	5	83.3%	
**Shock at admission**						0.7521
Yes	**35**	15	42.9%	20	57.1%	
No	**59**	22	37.3%	37	62.7%	
**Arterial treatment**						0.9858
Venous graft	**53**	10	18.9%	43	81.1%	
End-to-end anastomosis	**21**	4	19.0%	17	81.0%	
**Venous treatment**						0.3479
Ligation	**48**	13	27.1%	35	72.9%	
Venorrhaphy	**8**	1	12.5%	7	87.5%	
**Inappropriate surgical decision**						**0.0001** *
Present	**15**	14	93.3%	1	6.7%	
Absent	**79**	23	29.1%	56	70.9%	

* G test of independence; N: number of cases; %: percentage of cases.

Although reinterventions were more frequent when arterial and venous injuries were both present, there were no statistically significant differences in the common femoral vessels, superficial femoral vessels, or popliteal vessels (p = 0.4069; p = 0.1681; and p = 0.2593, respectively). Surgical reintervention was not statistically associated with a need for fasciotomy (p = 0.1571), with occurrence of fracture/luxation (p = 0.3543), with any specific mechanism of trauma (p = 0.9554), presence of hypovolemic shock at admission (p = 0.7521), or with the type of treatment employed for arterial (p = 0.9858) or venous (p = 0.3479) injuries (Table 5).

**Figure 3:**
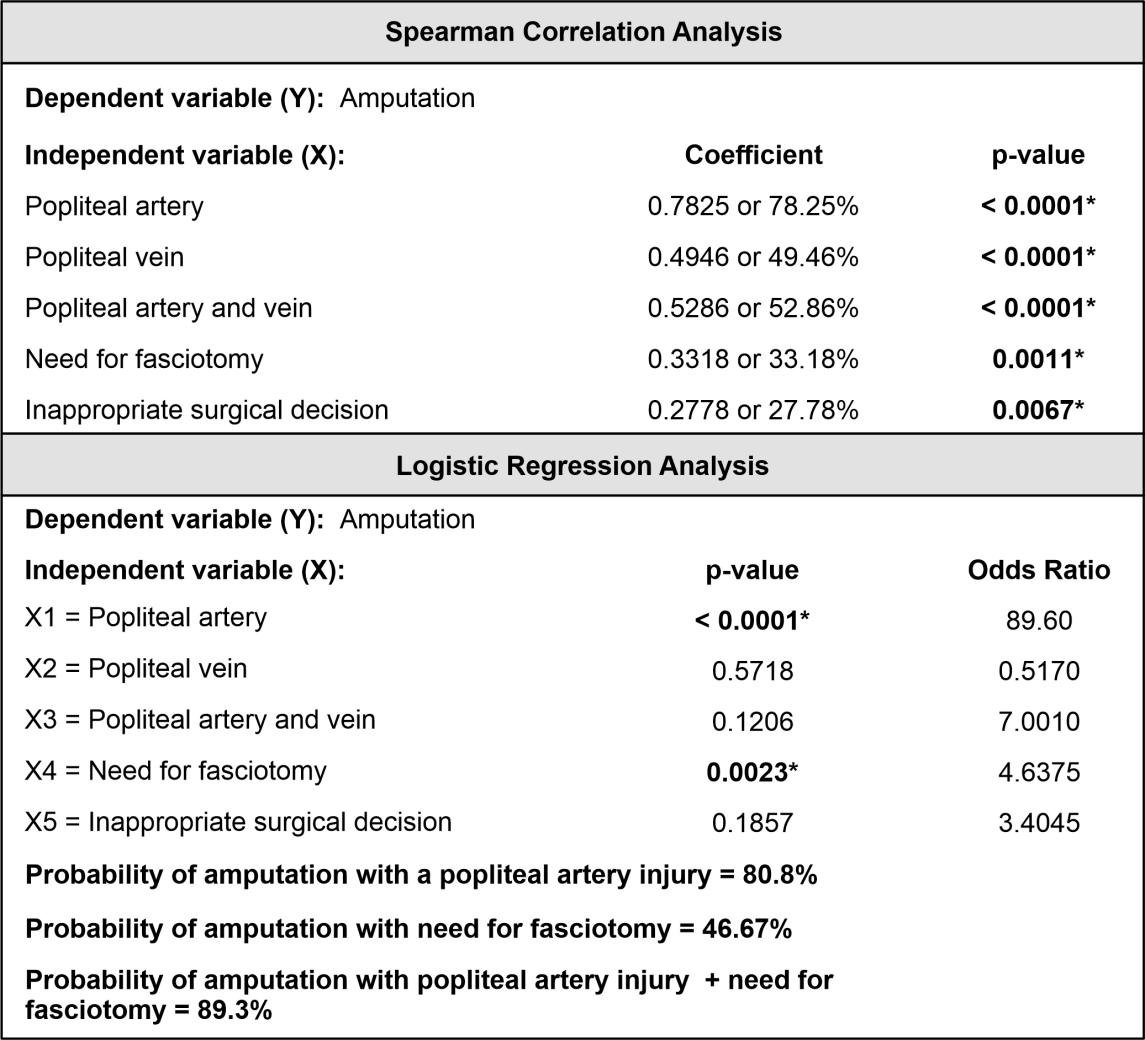
Spearman correlation analysis and logistic regression for the variable amputation. *Spearman's correlation coefficient.

The outcome “amputation” was statistically more frequent when the following variables were present: isolated popliteal artery injury (*p = 0.0334), presence of fracture or luxation (*p = 0.0003), need for fasciotomy (*p < 0.0001), venous ligation (*p = 0.0194), inappropriate surgical decisions (*p = 0.0110), and traffic accident as trauma mechanism (*p = 0.0002) (Table 6). However, when these variables were included in the logistic regression equation, the dependent relationship with the outcome amputation was only confirmed for popliteal artery injuries and need for fasciotomy: the probability of amputation for all popliteal artery injuries was 80.8% and the probability for those with popliteal artery injuries and a need for fasciotomy was 89.3% (Figure 3).

**Table 6:** Unfavorable outcome amputation and relationships with the other study variables.

**Variables**	**N**	**Amputation**				
		**Yes (n=24)**		**No (n=70)**		***p*-value**
**Superficial femoral (n=58)**						0.8270
Artery and vein	**32**	2	6.3%	30	93.8%	
Artery only	**24**	2	8.3%	22	91.7%	
Vein only	**2**	0	0.0%	2	100.0%	
**Popliteal (n=31)**						0.0334*
Artery and vein	**21**	14	66.7%	7	33.3%	
Artery only	**7**	7	100.0%	0	0.0%	
Vein only	**3**	1	33.3%	2	66.7%	
**Common femoral (n=14)**						0.4069
Artery and vein	**6**	1	16.7%	5	83.3%	
Artery only	**6**	0	0.0%	6	100.0%	
Vein only	**2**	0	0.0%	2	100.0%	
**Needed fasciotomy**						< 0.0001*
Yes	**30**	16	53.3%	14	46.7%	
No	**64**	8	12.5%	56	87.5%	
**Fracture and/or luxation**						0.0003*
Yes	**34**	16	47.1%	18	52.9%	
No	**60**	8	13.3%	52	86.7%	
**Trauma mechanism**						**0.0002** *
Gunshot	**70**	11	15.7%	59	84.3%	
Traffic accident	**18**	12	66.7%	6	33.3%	
Knife wound	**6**	1	16.7%	5	83.3%	
**Shock at admission**						0.7826
Yes	**35**	10	28.6%	25	71.4%	
No	**59**	14	23.7%	45	76.3%	
**Arterial treatment**						0.4981
Venous graft	**53**	14	26.4%	39	73.6%	
End-to-end anastomosis	**21**	4	19.0%	17	81.0%	
**Venous treatment**						0.0194*
Ligation	**48**	15	31.3%	33	68.8%	
Venorrhaphy	**8**	0	0.0%	8	100.0%	
**Inappropriate surgical decision**						0.0110*
Present	**15**	8	53.3%	7	46.7%	
Absent	**79**	16	20.3%	63	79.7%	

* G test of independence; N: number of cases; %: percentage of cases.

The variable “venous ligation” was analyzed separately for each of the different sites of vascular injury studied, revealing no statistically significant associations with the outcome amputation. Venous ligation was performed on superficial femoral (p = 0.5080), popliteal (p = 0.0930), or common femoral (p = 0.0712) veins (Table 7). The probability of death was not statistically associated with the need for fasciotomy (p = 0.4993), concomitant fracture or luxation (p = 0.5132), trauma mechanism (p = 0.1198), treatment of arterial (p = 0.3556) or venous (p = 0.1278) injuries, presence of shock at admission (p = 0.2404), or inappropriate surgical decisions (p = 0.6632). Combinations of arterial and venous injuries in the same patient were also not associated with death, irrespective of which vessels were injured (Table 8). The ISS for patients who died ranged from 13 to 41, with a mean of 21.67, whereas it was from 10 to 41 (mean of 17.07) among those who survived. However, this difference was not statistically significant.

**Table 7:** Use of venous ligation and relationship with progression to limb amputation.

**Variables**	**N**	**Amputation**				
		**Yes (n=24)**		**No (n=70)**		***p*-value**
**Superficial femoral vein (n=31)**						0.5080
With venous ligation	**25**	1	4.0%	24	96.0%	
Without venous ligation	**6**	0	0.0%	6	100.0%	
**Popliteal vein (n=24)**						0.0930
With venous ligation	**20**	14	70.0%	6	30.0%	
Without venous ligation	**4**	1	25.0%	3	75.0%	
**Common femoral vein (n=08)**						0.0712
With venous ligation	**6**	0	0.0%	6	100.0%	
Without venous ligation	**2**	1	50.0%	1	50.0%	

G test of independence; N: number of cases; %: percentage of cases.

**Table 8:** Unfavorable outcome death and relationships with the other study variables.

**Variables**	**N**	**Outcome of treatment**				
		**Survival (n=85)**		**Death (n=09)**		***p*-value**
**Superficial femoral (n=58)**						0.5442
Artery and vein	**32**	27	84.4%	5	15.6%	
Artery only	**24**	22	91.7%	2	8.3%	
Vein only	**2**	2	100.0%	0	0.0%	
**Popliteal (n=31)**						0.0814
Artery and vein	**21**	21	100.0%	0	0.0%	
Artery only	**7**	7	100.0%	0	0.0%	
Vein only	**3**	2	66.7%	1	33.3%	
**Common femoral (n=14)**						0.1717
Artery and vein	**6**	4	66.7%	2	33.3%	
Artery only	**6**	6	100.0%	0	0.0%	
Vein only	**2**	2	100.0%	0	0.0%	
**Needed fasciotomy**						0.4993
Yes	**30**	28	93.3%	2	6.7%	
No	**64**	57	89.1%	7	10.9%	
**Fracture and/or luxation**						0.5132
Yes	**34**	30	88.2%	4	11.8%	
No	**60**	55	91.7%	5	8.3%	
**Trauma mechanism**						0.1198
Gunshot	**70**	65	92.9%	5	7.1%	
Traffic accident	**18**	14	77.8%	4	22.2%	
Knife wound	**6**	6	100.0%	0	0.0%	
**Shock at admission**						0.2404
Yes	**35**	30	85.7%	5	14.3%	
No	**59**	55	93.2%	4	6.8%	
**Arterial treatment**						0.3556
Venous graft	**53**	47	88.7%	6	11.3%	
End-to-end anastomosis	**21**	20	95.2%	1	4.8%	
**Venous treatment**						0.1278
Ligation	**48**	41	85.4%	7	14.6%	
Venorrhaphy	**8**	8	100.0%	0	0.0%	
**Inappropriate surgical decision**						0.6632
Present	**15**	1	6.7%	14	93.3%	
Absent	**79**	8	10.1%	71	89.9%	

G test of independence; N: number of cases; %: percentage of cases.

## DISCUSSION

Vascular surgeons are increasingly called on to provide care at trauma centers, primarily to deal with ischemic limbs, control hemorrhages, and help during complex surgical exposures.^11,14,33^ However, inadequate training in vascular trauma can have a negative impact on the outcomes of these cases. Around 70% of traumatic vascular injuries involve the lower limbs^3,25^ and the superficial femoral artery is the vessel most often damaged.^1,34^ Injuries to the popliteal vessels are responsible for high amputation rates,^5,18,19,23,24^ underscoring the importance of studying prognostic factors associated with these injuries.

The deep femoral vessels are rarely injured, and it is known that their venous ligation is not a critical issues, whether for arterial perfusion or for venous drainage of the limb.^18^ For these reasons, injuries to these vessels were not included in this analysis. Patients with injuries to other sites that could confound the cause of death were also excluded from analysis of this outcome. All analyses were conducted with the sole objective of assessing the repercussions for patient prognosis of the vascular injuries studied. Only surgical reinterventions related to the vascular injuries were included in the analyses. Although all types of patients are subject to traumatic vascular injuries, the vast majority of such injuries involve young men,^1-5,7,11,12,33^ as was detected in our study.

The etiology of vascular trauma is not uniform. On the American and African continents, gunshot and knife wounds are the most common,^2,3^ as was observed in this sample. The superficial femoral vessels were the most often injured, confirming the literature.^1,8,17,33,34^ Injuries to the superficial femoral artery lead to limb loss in 7 to 13% of cases,^3,35^ also agreeing with our study, in which 8.3% of the patients with these injuries had amputations.

The popliteal artery was the second most frequently injured in this sample, with an amputation rate of 26%.^5^ All seven cases of isolated popliteal artery injury progressed to amputation, which can be explained by the small number of cases and the large distances the patients had to travel for treatment. Regarding the treatments used for arterial traumas, the literature describes autologous vein graft as the most common technique for repairing these injuries^18,23,27^ and it was used in 59.6% of these cases. When resection of the damaged arterial stumps permits end-to-end anastomosis without tension, this technique can be chosen^2,21,25^ - and it was the second most frequent strategy employed in the present sample (23.6% of cases). Treatments described for venous injuries include venorrhaphy, end-to-end anastomosis, graft interposition, and venous ligation.^12,33^ In the present sample, venous ligation was employed in 81.4% of cases, followed by venorrhaphy, in 13.6% of the patients.

Vascular injuries of the extremities may occur in conjunction with skeletal traumas or traumas of other areas^1,2,5,18,25,27^ and 69.2% of the cases in this sample had additional injuries. Additional injuries are more common in blunt trauma cases and increase the risk of amputation.^1,5,18,25-27^ The results of the present study bear out this reasoning, since a statistically significant association was detected between blunt trauma and limb amputation. These injuries are also more likely to result in compartment syndrome, because of combinations of fractures and vascular injuries,^1,5,18,21,26,27^ and our results also demonstrated that a need for fasciotomy was associated with a higher frequency of amputation.

Several studies have already shown that rapid transport, enabling timely hospital care, is determinant for better outcomes^3,14-16,18,19^ and that management of hypovolemic shock and early reperfusion of the injured limb are the pillars for treating extremities’ vascular traumas.^1,14,18^ This study reflects this situation and the significant proportion of patients who were already in shock at admission (37.2%) is linked to the fact that the hospital where the study was conducted is responsible for an area of 1,248,000 km^2^, where air ambulance rescue is often unavailable.^1,36^ Therefore, approximately 50% of the patients had to be transported more than 100 km by land and/or river before receiving care, negatively affecting the clinical outcomes of this study population.

One of the most contentious prognostic factors discussed in the literature is the fact that venous ligation possibly predisposes to limb amputation. Many studies have already confirmed this correlation.^14,29^ However, many contemporary authors now suggest that venous ligation does not actually increase this risk and that repairing traumatized veins may increase the risk of thromboembolism.^28,29^ Our results are in line with these authors, since no statistically significant associations were found between venous ligation and amputation in injuries to any of the three studied topographies.

No prior studies were found that have attempted to assess the impact of inappropriate surgical decisions on the outcomes of vascular injury victims. This study found evidence of cases in which the chosen techniques were contrary to classical principles. There were cases of gunshot wounds in which the arterial injury was only treated with thrombectomy followed by arteriorrhaphy, without resecting the traumatized segment. This strategy maintains traumatized endothelium, predisposing to thrombosis and consequent ischemia.^25,28,37,38^ Cases were also detected in which there were simultaneous arterial and venous injuries and the vein was treated by venous ligation and the surgeon decided to remove the great saphenous vein from the traumatized limb for arterial reconstruction. However, the classical recommendation is to use the contralateral saphenous vein, because reduced drainage via the superficial veinous system is prejudicial to compensation of venous return in the limb that undergoes deep veinous ligation, causing congestion, compartment syndrome, and irreversible ischemia.^18,25,37,39^

The severity of the case does not justify using these maneuvers, which literature classically describes as inappropriate.^37-39^ If it is not possible to the correct techniques because of hemodynamic instability, lack of necessary materials, or other reasons, it is recommended that “damage control” strategies capable of having a positive impact on prognosis of the traumatized limb should be employed.^2,3,10,17,25^ The fact that 37.2% of the patients were admitted in hypovolemic shock contrasts with the sporadic description of damage control techniques in this sample, such as a temporary vascular shunt, a tool surgeons who deal with these traumas should master.^2,3,9,11,17,25^

All of the cases analyzed in this study were operated by vascular surgeons. Despite this, as demonstrated, failure to observe traditional concepts regarding vascular management trauma management was relatively frequent and was associated with unfavorable outcomes. The reasons for these events are probably multifactorial: team members may have been heterogeneous in terms of their training in vascular trauma during medical residency and their personal accumulated experience, emphasizing the need for specific attention to treatment of traumatic vascular injuries, with emphasis on damage control strategies.

**Figure 4:**
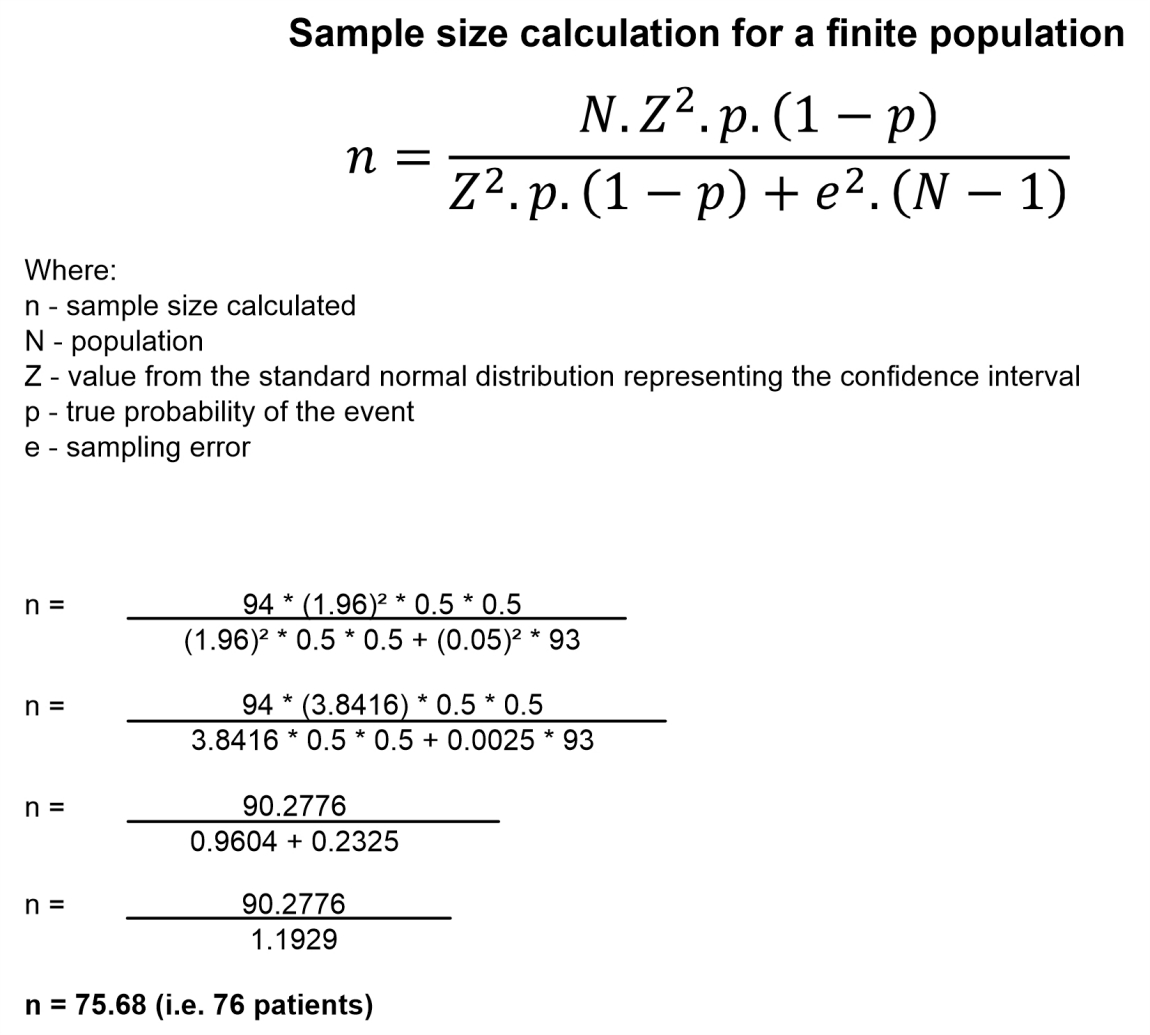
Sample size calculation.

The literature describes elevated ISS scores as an important prognostic factor of survival among vascular trauma victims.^6,18,22,25^ However, no statistically significant difference was detected between the ISS of fatal trauma victims and the scores of those who survived, probably because of the sample size, which also explains why shock at hospital admission, described as a factor linked to higher mortality,^2,6,31,32^ was also not statistically significantly associated with death. Although we analyzed all cases meeting the inclusion criteria that were treated during the study period, a sample size calculation for a finite population was performed post hoc, showing that for this population a sample of 76 patients would be considered significant (Figure 4). Trauma is a peculiar field of study. Cases have heterogeneous mechanisms and outcomes, and it is common that the severity of clinical status precludes an ideal recording of variables.

Limitations of this study include its retrospective design, with incomplete medical records and surgical descriptions that were not always precise. Additionally, although the number of cases did exceed the minimum size calculated for a significant sample, it is possible that conducting prospective multicenter projects with larger samples could mitigate these limitations. The authors suggest including assessment of inappropriate surgical decisions among future studies’ variables, since this preliminary research suggests that this could be an important factor associated with unfavorable prognosis among these patients.

## CONCLUSIONS

Most of the victims of vascular injuries to the femoropopliteal segment are men of approximately 30 years old, victims of gunshot wounds. Injuries to the superficial femoral vessels were more frequent than injuries to the popliteal vessels, while the common femoral vessels were the least often involved. Concomitant injuries to non-vascular structures were common, of which fractures were the most frequent. Venous grafting was the treatment most used for arterial traumas, and venous ligation was most often used for venous injuries. Blunt traumas, caused by traffic accidents, were more often associated with limb amputation, when compared to the other trauma mechanisms. Venous ligation did not increase the probability of limb amputation. Inappropriate surgical decisions resulted in a higher probability of reinterventions. Popliteal artery injuries and a need for fasciotomy increased the limb amputation probability. None of the studied variables had statistically significant correlation with death.
