# Supplementary material for: Prognostic factors for femoropopliteal vascular injuries: surgical decisions matter
Source: J Vasc Bras. 2023 Nov 13;22:e20230050. doi: 10.1590/1677-5449.202300502 (PMC10647908; doi:10.1590/1677-5449.202300502)
Supplement: Translation

# Translation: Fatores prognósticos em traumatismos vasculares do segmento femoropoplíteo: decisões intraoperatórias são importantes

**DOI:** 10.1590/1677-5449.202300501

**Authors:** Adenauer Marinho de Oliveira Góes, Fernanda Beatriz Araújo de Albuquerque, Matheus Oliveira Feijó, Flávia Beatriz Araújo de Albuquerque, Luciana Roberta do Vale Corrêa, Mariseth Carvalho de Andrade

**Affiliations:** 1 Universidade Federal do Pará - UFPA, Belém, PA, Brasil.; 2 Universidade Federal do Paraná - UFPR, Hospital das Clinicas - HC, Curitiba, PR, Brasil.; 3 Força Aérea Brasileira - FAB, Belém, PA, Brasil.; 4 Exército Brasileiro - EB, Macapá, AP, Brasil.; 5 Hospital Metropolitano de Urgência e Emergência - HMUE, Ananindeua, PA, Brasil.; 6 Fundação Santa Casa de Misericórdia do Pará - FSCMPA, Belém, PA, Brasil.

**Keywords:** lesões do sistema vascular, membros inferiores, procedimentos cirúrgicos vasculares, prognóstico

## Abstract

**Contexto:**

O trauma vascular acomete frequentemente os membros inferiores; entretanto, ainda há dúvidas sobre quais fatores levam a desfechos desfavoráveis.

**Objetivos:**

Determinar o perfil das vítimas de traumatismo femoropoplíteo, o tratamento utilizado e fatores relacionados a desfechos desfavoráveis.

**Métodos:**

Estudo retrospectivo, baseado em prontuários de pacientes operados entre 2017 e 2021. Foram analisados: sexo, idade, distância percorrida para atendimento, mecanismo de trauma, presença de choque hipovolêmico, lesões associadas, tratamento, realização de fasciotomia, decisões intraoperatórias inadequadas e índice de severidade de trauma. Necessidade de reintervenção, amputação e óbito foram considerados desfechos desfavoráveis. Foram utilizadas análises univariadas, bivariadas e regressão logística.

**Resultados:**

Noventa e quatro pacientes foram selecionados, sendo 83% homens, com idade média de 30,8 anos. Lesões arteriais e venosas simultâneas ocorreram em 57,5% dos casos; vasos femorais superficiais foram mais acometidos (61,7%), e mecanismos penetrantes, mais prevalentes (80,9%). Lesões arteriais foram frequentemente tratadas com enxerto venoso (59,6%), e lesões venosas foram submetidas à ligadura (81,4%). Em 15% houve decisões cirúrgicas inadequadas, sendo o uso da safena magna ipsilateral para reconstrução arterial a mais comum. Ocorreram desfechos desfavoráveis em 44,7% dos casos; em 21,3%, foi necessária reintervenção; amputação em 25,5%; e ocorreu óbito em 9,5% dos pacientes.

**Conclusões:**

As lesões acometeram principalmente homens jovens, vítimas de ferimento por arma de fogo. Vasos femorais superficiais foram os mais lesados, e traumatismos não vasculares concomitantes foram frequentes, principalmente fraturas. Decisões cirúrgicas inadequadas aumentaram em 34 vezes a necessidade de reintervenções. Necessidade de realização de fasciotomia, presença de fratura/luxação, mecanismo contuso de trauma e lesão de artéria poplítea aumentaram o risco de amputação.

## INTRODUÇÃO

Embora acometa principalmente homens jovens^1-8^ , pacientes de todas as idades e gêneros podem sofrer lesões vasculares traumáticas. A frequência desses traumatismos tem sido crescente, estimando-se que cerca de 6% dos traumatizados civis apresentem lesão vascular^2^ , sendo que em militares essas lesões representem até 17,6% dos traumas^2-4,8-12^ .

O acometimento de vasos dos membros é comum e acarreta riscos de óbito e amputação^4,5^ . Nos casos de traumatismos vasculares dos membros, muitos fatores influenciam o desenvolvimento de desfechos clínicos desfavoráveis. Um dos primeiros a ser estabelecido, há mais de 70 anos^13^ , é o tempo de isquemia, que se correlaciona diretamente com a probabilidade de amputação do membro^14-19^ .

A topografia e o mecanismo de lesão também interferem no prognóstico^1,20,21^ . Sabe-se que a hemorragia associada a lesões dos vasos femorais comuns pode levar a uma mortalidade de até 8%^1,22^ , e que lesões da artéria poplítea, onde a circulação colateral é menos desenvolvida, apresentam taxas de amputação de até 26%^5,18,19,23,24^ . Também é consenso que traumatismos contusos têm pior prognóstico em relação aos penetrantes^1,18,21,25-27^ .

Entretanto, há questões ainda não resolvidas: no cenário de lesões venosas, referências da literatura se dividem quanto ao fato da ligadura aumentar ou não o risco de amputação^14,28,29^ , e não foram localizados estudos avaliando sobre como decisões cirúrgicas inadequadas afetam o prognóstico dos pacientes. Esta pesquisa objetivou determinar o perfil das vítimas de traumatismo vascular femoropoplíteo, os mecanismos de trauma e as técnicas empregadas em seu tratamento, e verificar quais fatores influenciaram no desenvolvimento de desfechos clínicos desfavoráveis.

## MÉTODOS

Pesquisa aprovada pelo Comitê de Ética Institucional (CAAE 2114919.8.0000.5169, parecer 4928779). Trata-se de estudo retrospectivo analítico baseado no levantamento de prontuários eletrônicos de janeiro de 2017 a dezembro de 2021. Todos os prontuários contendo os termos “femoral” ou “poplítea” foram identificados e revisados. Foram incluídos pacientes de ambos os sexos, com idade superior a 16 anos, submetidos a tratamento cirúrgico por lesões traumáticas de artérias ou veias femorais comuns, femorais superficiais ou poplíteas provocadas por qualquer mecanismo.

Casos operados em outros hospitais e posteriormente transferidos para reavaliação, aqueles submetidos à amputação primária ou com amputação traumática do membro ou apresentando lesões potencialmente fatais concomitantes, que pudessem atuar como viés confundidor para análise dos desfechos, foram excluídos (traumatismos cardíacos, de outros vasos sanguíneos, de vísceras abdominais maciças, fraturas de bacia, hemotórax maciço, traumatismos cranioencefálicos ou outros que pudessem ocasionar o óbito precoce do paciente), assim como aqueles cujos prontuários apresentaram informações insuficientes. Foram analisados sexo e idade dos pacientes, agrupados em: menor que 30 anos, entre 30 e 39 anos e maior que 40 anos. A distância entre a localidade onde o trauma ocorreu e o hospital foi medida pelo aplicativo Google Maps®, e os intervalos foram classificados como inferior ou superior a 100 km.

Os mecanismos de trauma foram classificados como penetrantes (arma de fogo [FAF] e arma branca [FAB]) ou contusos (acidentes de trânsito, quedas e outros mecanismos, e a estrutura vascular acometida foi registrada. Choque hipovolêmico na admissão foi definido como pressão arterial sistólica menor que 90 mmHg ou frequência cardíaca maior que 100 batimentos por minuto, e o índice de gravidade de trauma (*Injury Severity Score* [ISS])^30^ foi calculado^31,32^ .

Lesões não vasculares associadas foram classificadas como lesões esqueléticas, de tórax, de abdome/pelve e de cabeça/pescoço. Lesões arteriais e venosas foram classificadas como secção, trombose, pseudoaneurisma e fístula arteriovenosa. As técnicas utilizadas no tratamento foram categorizadas em enxerto venoso, enxerto com prótese, anastomose término-terminal, trombectomia, arteriorrafia, venorrafia, *patch*, *shunt* temporário, ligadura e anticoagulação. Pesquisou-se também a realização de fasciotomias.

A utilização da veia safena magna ipsilateral na lesão para reconstrução vascular, a arteriorrafia primária e a trombectomia seguida de arteriorrafia (sem desbridamento parietal/ressecção de segmento lesionado), além da não detecção de lesões durante a exploração vascular, foram classificadas como “decisões cirúrgicas inadequadas”. Considerou-se como reintervenção cirúrgica a necessidade de nova intervenção do cirurgião vascular para desbridamentos, fasciotomias em segundo tempo ou ampliação de fasciotomias realizadas inicialmente. Necessidade de reintervenção, evolução para amputação e óbito foram consideradas como desfechos desfavoráveis e sua ocorrência foi correlacionada com as variáveis acima descritas.

Para análise estatística, foram utilizados os *softwares* Microsoft Office Excel® 2016 e BioEstat® 5.4. A estatística analítica foi utilizada para avaliar resultados das variáveis categóricas, os testes G e qui-quadrado, para análises univariadas, e teste G para comparações bivariadas. O teste de correlação de Spearman foi usado para as variáveis significantes em relação aos desfechos desfavoráveis, e, após identificação das variáveis correlacionadas, a equação de regressão logística possibilitou o cálculo das probabilidades em relação à variável dependente. Foi adotado o nível de significância α = 0,05 ou 5%.

## RESULTADOS

O levantamento inicial detectou 1.057 prontuários; após aplicação dos critérios de inclusão e exclusão, 94 pacientes foram selecionados. O processo de seleção para composição da amostra está apresentado na Figura 1. A amostra foi composta por 78 pacientes do sexo masculino (83%) e 16 do sexo feminino (17%) (*p < 0,0001). A idade dos pacientes variou entre 16 e 70 anos, com média de 30,8 anos; 54,3% dos pacientes (51/94) tinham menos de 30 anos (*p < 0,0001). O trajeto até o atendimento foi estabelecido em 94,7% dos casos (89/94), tendo sido de até 100 km para 47,9% (45/94), enquanto 46,8% dos pacientes (44/94) percorreram mais de 100 km (p = 0,9156). Lesões associadas foram detectadas em 55,3% dos casos (52/94) (p = 0,3023). Entre essas, os traumas esqueléticos foram os mais comuns, em 69,2% dos pacientes (*p = 0,0055) (Tabela 1).

**Figura 1:** *Flowchart* da composição da amostra.

**Tabela 1:** Variáveis sociodemográficas e lesões associadas.

**Variáveis**	**N**		**%**		**p-valor**
**Sexo**					< 0,0001*
Feminino	16		17,0%		
Masculino*	78		83,0%		
**Faixa etária**					< 0,0001*
< 30*	51		54,3%		
30 a 39	24		25,5%		
> = 40	19		20,2%		
**Mínima/média/máxima**		**16 / 30,8 / 70 anos**			
**Distância (Km)**					0,9156
Até 100*	45		47,9%		
Acima de 100	44		46,8%		
Sem registro	5		5,3%		
**Lesões associadas**					0,3023
Presença	52		55,3%		
Ausência	42		44,7%		
**Estrutura lesada**	**52**||		**55,3%**		**0,0055** †
Esqueleto apendicular*‡	36¶		69,2%		
Outras lesões vasculares§	13		25,0%		
Abdome/pelve	9		17,3%		
Tórax	3		5,8%		
Cabeça/pescoço	1		1,9%		

* Teste qui-quadrado aderência;

† Teste G aderência;

N: número de casos; %: porcentagem de casos;

‡ Fratura: calcâneo (1), tíbia (15), fíbula (2), fêmur (20), carpo (1), radio (3), ulna (2), úmero (2); em relação à luxação, foram detectados: joelho (5) e quadril (2);

§ outras lesões vasculares: artéria femoral profunda (4), artéria tibial anterior (2), vasos pudendos (1), tronco tibiofibular (2), veia ilíaca externa (1), veia femoral profunda (1), artéria genicular (1);

|| N corresponde ao número de pacientes com lesões de outras estruturas; houve pacientes que apresentaram lesões simultâneas em mais de uma estrutura, além das lesões vasculares pesquisadas;

¶ Alguns pacientes apresentaram lesão de mais de uma estrutura do esqueleto apendicular.

Lesões arteriais e venosas simultâneas foram estatisticamente mais comuns do que lesões arteriais ou venosas isoladas (*p < 0,0001), representando 57,5% (54/94) dos casos, e os vasos mais acometidos foram os femorais superficiais (61,7%) (*p = 0,0233). Uma lesão arterial foi identificada em 94,7% (89/94) (*p < 0,0001) da amostra. A artéria mais acometida foi a femoral superficial (62,9%) (*p = 0,0197), seguida pelas artérias poplítea (30,3%) e femoral comum (11,2%). Lesões venosas ocorreram em 62,8% (59/94) dos casos (*p = 0,0039), sendo a femoral superficial lesada em 52,5% dos casos (*p = 0,0013), seguida por poplítea (40,7%) e femoral comum (13,6%) (Tabela 2).

**Tabela 2:** Estrutura vascular acometida.

**Variáveis**	**N**	**%**	**p-valor**
**Estrutura vascular acometida**			**< 0,0001** *
Artéria e veia*	54	57,50%	
Somente artéria	35	37,20%	
Somente veia	5	5,30%	
**Vasos lesados**			
Femorais superficiais*	58	61,70%	0,0233*
Poplíteos	31	33,00%	
Femorais comuns	14	14,90%	
Artérias lesadas*	**89^†^**	**94,70%**	< 0,0001*
Femoral superficial*	56	62,90%	0,0197*
Poplítea	27	30,30%	
Femoral comum	10	11,20%	
Veias lesadas^*^	**59^†^**	**62,80%**	0,0039*
Femoral superficial*	31	52,50%	0,0013*
Poplítea	24	40,70%	
Femoral comum	8	13,60%	

* Teste qui-quadrado aderência; N: número de casos; %: porcentagem de casos;

† N corresponde ao número de pacientes que apresentaram ao menos uma lesão de uma das artérias ou veias pesquisadas; houve pacientes que apresentaram mais de uma lesão arterial/venosa pesquisada.

Mecanismos penetrantes de trauma prevaleceram, ocorrendo em 80,9% (76/94) (*p < 0,0001). Entre esses, predominaram FAF (92,1%) sobre FAB (7,9%) (*p < 0,0001). Todos os casos de trauma contuso foram provocados por acidentes de trânsito.

A maior parte das lesões, tanto arteriais (52,8%) quanto venosas (40,2%), foi classificada como secção (parcial/total) dos vasos (*p < 0,0001). Lesões arteriais foram mais frequentemente tratadas com enxerto venoso (59,6%) ou anastomose término-terminal (23,6%), enquanto a maioria das lesões venosas foi submetida à ligadura (81,4%) ou à venorrafia (13,6%) (*p < 0,0001) (Tabela 3).

**Tabela 3:** Características das lesões vasculares e tratamentos empregados.

**Variáveis**	**N**	**%**	**p-valor**
**Mecanismo do trauma**			< 0,0001*
**Penetrante** *	**76**	**80,9%**	
Arma de fogo	70	92,1%	
Arma branca	6	7,9%	
**Contusos**	**18**	**19,1%**	
Acidente de trânsito	18	100,0%	
**Descrição da lesão arterial**	**89** †		< 0,0001*
Secção parcial/total*	47	52,8%	
Trombose	16	18,0%	
Pseudoaneurisma	7	7,9%	
Fístula arteriovenosa	6	6,7%	
Não registrado	18	20,2%	
**Tratamento arterial**			< 0,0001*
Enxerto venoso*	53	59,6%	
Anastomose término-terminal	21	23,6%	
Trombectomia a Fogarty	5	5,6%	
Ligadura	4	4,5%	
Arteriorrafia	3	3,4%	
Enxerto com prótese	1	1,1%	
*Patch*	1	1,1%	
*Shunt* arterial	1	1,1%	
**Descrição da lesão venosa**	**59^†^**		< 0,0001*
Secção parcial/total*	29	49,2%	
Fístula arteriovenosa	6	10,2%	
Trombose	1	1,7%	
Não registrado	23	39,0%	
**Tratamento venoso**			< 0,0001*
Ligadura*	48	81,4%	
Venorrafia	8	13,6%	
Anticoagulação	1	1,7%	
Não registrado	2	3,4%	

* Teste G aderência; N: número de casos; %: porcentagem de casos;

† N corresponde ao número de pacientes que apresentaram ao menos uma lesão de uma das artérias ou veias pesquisadas; houve pacientes que apresentaram mais de uma lesão arterial/venosa pesquisada.

Na maioria dos casos, a revisão da descrição cirúrgica não demonstrou decisões cirúrgicas inadequadas. Essas ocorreram em 15,9% dos casos (15/94) (*p < 0,0001). A situação mais comum foi o uso da safena magna do membro lesionado para enxerto venoso, em 10 pacientes; outras situações envolveram arteriorrafia e trombectomia sem a ressecção do segmento traumatizado e casos em que a exploração vascular não detectou lesões presentes e que foram diagnosticadas tardiamente, após a piora do quadro clínico. Desfechos desfavoráveis ocorreram em 44,7% dos casos (42/94) (p = 0,1891), sendo 38,1% (16/42) somente amputação, 23,8% (10/42) somente reintervenção, e 11,9% (5/42) somente óbito. Em 11 casos, houve associação entre tais desfechos (Tabela 4).

**Tabela 4:** Desfechos desfavoráveis e decisões cirúrgicas inadequadas.

**Variáveis**	**N**	**%**	**p-valor**
**Decisões cirúrgicas inadequadas**			< 0,0001*
Presença	15	16,0%	
Ausência*	79	84,0%	
**Desfecho desfavorável**			0,1891
Sim	42	44,7%	
Não	52	55,3%	
Tipo de desfecho desfavorável*			0,5465
Amputação	24	25,5%	
Reintervenção	20	21,3%	
Óbito	9	9,6%	
**Associação de desfechos desfavoráveis**	**42**		0,0255*
Somente amputação*	16	38,1%	
Somente reintervenção	10	23,8%	
Somente óbito	5	11,9%	
Amputação + reintervenção	7	16,7%	
Reintervenção + óbito	3	7,1%	
Amputação + óbito	1	2,4%	

* Teste G aderência; N: número de casos; %: porcentagem de casos.

Quando analisada a necessidade de reintervenção, detectou-se associação estatisticamente significativa com decisões cirúrgicas inadequadas (*p = 0,0001). Quando não foram detectadas essas associações, a taxa de reintervenção foi 29,1%, e quando presentes, foi de 93,3% (Tabela 5), correspondendo a um aumento de 34,1 vezes na probabilidade de uma reintervenção cirúrgica ser necessária (Figura 2). As reintervenções mais frequentes corresponderam a desbridamentos (10), fasciotomia ou ampliação desta (8), enxerto venoso (5), trombectomia (4), ligadura arterial (1), e correção de pseudoaneurisma (1).

**Tabela 5:** Desfecho desfavorável reintervenção e relação com as diversas variáveis pesquisadas.

**Variáveis**	**N**	**Reintervenção cirúrgica**				
		**Sim (n=20)**		**Não (n=74)**		**p-valor**
**Vasos femorais superficiais (n=58)**						0,1681
Artéria e veia	**32**	8	25,0%	24	75,0%	
Somente artéria	**24**	2	8,3%	22	91,7%	
Somente veia	**2**	0	0,0%	2	100,0%	
**Vasos poplíteos (n=31)**						0,2593
Artéria e veia	**21**	8	38,1%	13	61,9%	
Somente artéria	**7**	2	28,6%	5	71,4%	
Somente veia	**3**	0	0,0%	3	100,0%	
**Vasos femorais comuns (n=14)**						0,4069
Artéria e veia	**6**	1	16,7%	5	83,3%	
Somente artéria	**6**	0	0,0%	6	100,0%	
Somente veia	**2**	0	0,0%	2	100,0%	
**Necessidade de fasciotomia**						0,1571
Sim	**30**	9	30,0%	21	70,0%	
Não	**64**	11	17,2%	53	82,8%	
**Fratura e/ou luxação**						0,3543
Sim	**34**	9	26,5%	25	73,5%	
Não	**60**	11	18,3%	49	81,7%	
**Mecanismo de trauma**						0,9554
Arma de fogo	**70**	15	21,4%	55	78,6%	
Acidente de trânsito	**18**	4	22,2%	14	77,8%	
Arma branca	**6**	1	16,7%	5	83,3%	
**Choque na admissão**						0,7521
Sim	**35**	15	42,9%	20	57,1%	
Não	**59**	22	37,3%	37	62,7%	
**Tratamento arterial**						0,9858
Enxerto venoso	**53**	10	18,9%	43	81,1%	
Anastomose término-terminal	**21**	4	19,0%	17	81,0%	
**Tratamento venoso**						0,3479
Ligadura	**48**	13	27,1%	35	72,9%	
Venorrafia	**8**	1	12,5%	7	87,5%	
**Decisão cirúrgica inadequada**						**0,0001** *
Presença	**15**	14	93,3%	1	6,7%	
Ausência	**79**	23	29,1%	56	70,9%	

* Teste G independência; N: número de casos; %: porcentagem de casos.

**Figura 2:** Análise de correlação de Spearman e regressão logística para variável reintervenção. *Teste de Correlação de Spearman.

Embora reintervenções tenham sido mais frequentes quando lesões arteriais e venosas ocorreram simultaneamente, tanto em vasos femorais comuns, femorais superficiais e poplíteos, não houve diferença estatisticamente significativa (p = 0,4069; p = 0,1681; e p = 0,2593, respectivamente). A necessidade de reintervenção cirúrgica também não esteve estatisticamente associada à necessidade de fasciotomia (p = 0,1571), à ocorrência de fratura/luxação (p = 0,3543), a determinado mecanismo de trauma (p = 0,9554), à presença de choque hipovolêmico na admissão (p = 0,7521) e ao tipo de tratamento empregado para as lesões arteriais (p = 0,9858) ou venosas (p = 0,3479) (Tabela 5).

O desfecho “amputação” foi estatisticamente mais frequente quando as seguintes variáveis estiveram presentes: lesão isolada de artéria poplítea (*p = 0,0334), presença de fratura ou luxação (*p = 0,0003), necessidade de fasciotomia (*p < 0,0001), ligadura venosa (*p = 0,0194), decisões cirúrgicas inadequadas (*p = 0,0110) e acidente de trânsito como mecanismo de trauma (*p = 0,0002) (Tabela 6). Entretanto, quando essas variáveis foram incluídas na equação de regressão logística, a relação de dependência com o desfecho amputação foi confirmada apenas para a lesão de artéria poplítea e a necessidade de fasciotomia: na lesão de artéria poplítea, a probabilidade de amputação foi de 80,8%, e especificamente entre aqueles com lesões da artéria poplítea e necessidade de fasciotomia, a probabilidade desse desfecho foi de 89,3% (Figura 3).

**Tabela 6:** Desfecho desfavorável amputação e relação com as diversas variáveis pesquisadas.

**Variáveis**	**N**	**Amputação**				
		**Sim (n=24)**		**Não (n=70)**		**p-valor**
**Femoral superficial (n=58)**						0,827
Artéria e veia	**32**	2	6,30%	30	93,80%	
Somente artéria	**24**	2	8,30%	22	91,70%	
Somente veia	**2**	0	0,00%	2	100,00%	
**Poplítea (n=31)**						0,0334^*^
Artéria e veia	**21**	14	66,70%	7	33,30%	
Somente artéria	**7**	7	100,00%	0	0,00%	
Somente veia	**3**	1	33,30%	2	66,70%	
**Femoral comum (n=14)**						0,4069
Artéria e veia	**6**	1	16,70%	5	83,30%	
Somente artéria	**6**	0	0,00%	6	100,00%	
Somente veia	**2**	0	0,00%	2	100,00%	
**Necessidade de fasciotomia**						**< 0,0001** *
Sim	**30**	16	53,30%	14	46,70%	
Não	**64**	8	12,50%	56	87,50%	
**Fratura e/ou luxação**						0,0003*
Sim	**34**	16	47,10%	18	52,90%	
Não	**60**	8	13,30%	52	86,70%	
**Mecanismo de trauma**						0,0002*
Arma de fogo	**70**	11	15,70%	59	84,30%	
Acidente de trânsito	**18**	12	66,70%	6	33,30%	
Arma branca	**6**	1	16,70%	5	83,30%	
**Choque na admissão**						0,7826
Sim	**35**	10	28,60%	25	71,40%	
Não	**59**	14	23,70%	45	76,30%	
**Tratamento arterial**						0,4981
Enxerto venoso	**53**	14	26,40%	39	73,60%	
Anastomose término-terminal	**21**	4	19,00%	17	81,00%	
**Tratamento venoso**						0,0194*
Ligadura	**48**	15	31,30%	33	68,80%	
Venorrafia	**8**	0	0,00%	8	100,00%	
**Decisão cirúrgica inadequada**						0,0110*
Presença	**15**	8	53,30%	7	46,70%	
Ausência	**79**	16	20,30%	63	79,70%	

* Teste G independência; N: número de casos; %: porcentagem de casos.

**Figura 3:** Análise de correlação de Spearman e regressão logística para variável amputação. *Teste de Correlação de Spearman.

A análise da variável “ligadura venosa”, feita separadamente para cada uma das topografais de lesão vascular estudadas, não demonstrou associação estatisticamente significativa com o desfecho amputação, tendo sido a ligadura realizada nas veias femoral superficial (p = 0,5080), poplítea (p = 0,0930) ou femoral comum (p = 0,0712) (Tabela 7). A probabilidade de óbito não foi estatisticamente associada à necessidade de fasciotomia (p = 0,4993), concomitância de fratura ou luxação (p = 0,5132), mecanismo de trauma (p = 0,1198), tratamento das lesões arteriais (p = 0,3556) ou venosas (p = 0,1278), presença de choque na admissão (p = 0,2404) ou decisões cirúrgicas inadequadas (p = 0,6632). A ocorrência de lesões arterial e venosa simultâneas também não esteve associada ao óbito, independentemente dos vasos traumatizados (Tabela 8). O *ISS* dos pacientes que evoluíram a óbito variou entre 13 e 41, com média de 21,67, e entre os pacientes que sobreviveram, esse variou entre 10 e 41 (média de 17,07). Entretanto, essa diferença não foi estatisticamente significativa.

**Tabela 7:** Uso da ligadura venosa e relação com evolução para amputação do membro.

**Variáveis**	**N**	**Amputação**				
		**Sim (n=24)**		**Não (n=70)**		**p-valor**
**Veia femoral superficial (n=31)**						0,508
Com ligadura	**25**	1	4,00%	24	96,00%	
Sem ligadura	**6**	0	0,00%	6	100,00%	
**Veia poplítea (n=24)**						0,093
Com ligadura	**20**	14	70,00%	6	30,00%	
Sem ligadura	**4**	1	25,00%	3	75,00%	
**Veia femoral comum (n=08)**						0,0712
Com ligadura	**6**	0	0,00%	6	100,00%	
Sem ligadura	**2**	1	50,00%	1	50,00%	

Teste G independência; N: número de casos; %: porcentagem de casos.

**Tabela 8:** Desfecho desfavorável óbito e relação com as diversas variáveis pesquisadas.

**Variáveis**	**N**	**Desfecho do tratamento**				
		**Vivo (n=85)**		**Óbito (n=09)**		**p-valor**
**Femoral superficial (n=58)**						0,5442
Artéria e veia	**32**	27	84,40%	5	15,60%	
Somente artéria	**24**	22	91,70%	2	8,30%	
Somente veia	**2**	2	100,00%	0	0,00%	
**Poplítea (n=31)**						0,0814
Artéria e veia	**21**	21	100,00%	0	0,00%	
Somente artéria	**7**	7	100,00%	0	0,00%	
Somente veia	**3**	2	66,70%	1	33,30%	
**Femoral comum (n=14)**						0,1717
Artéria e veia	**6**	4	66,70%	2	33,30%	
Somente artéria	**6**	6	100,00%	0	0,00%	
Somente veia	**2**	2	100,00%	0	0,00%	
**Necessidade de fasciotomia**						0,4993
Sim	**30**	28	93,30%	2	6,70%	
Não	**64**	57	89,10%	7	10,90%	
**Fratura e/ou luxação**						0,5132
Sim	**34**	30	88,20%	4	11,80%	
Não	**60**	55	91,70%	5	8,30%	
**Mecanismo de trauma**						0,1198
Arma de fogo	**70**	65	92,90%	5	7,10%	
Acidente de trânsito	**18**	14	77,80%	4	22,20%	
Arma branca	**6**	6	100,00%	0	0,00%	
**Choque na admissão**						0,2404
Sim	**35**	30	85,70%	5	14,30%	
Não	**59**	55	93,20%	4	6,80%	
**Tratamento arterial**						0,3556
Enxerto venoso	**53**	47	88,70%	6	11,30%	
Anastomose término-terminal	**21**	20	95,20%	1	4,80%	
**Tratamento venoso**						0,1278
Ligadura	**48**	41	85,40%	7	14,60%	
Venorrafia	**8**	8	100,00%	0	0,00%	
**Decisão cirúrgica inadequada**						0,6632
Presença	**15**	1	6,70%	14	93,30%	
Ausência	**79**	8	10,10%	71	89,90%	

Teste G independência; N: número de casos; %: porcentagem de casos.

## DISCUSSÃO

A atuação de cirurgiões vasculares em centros de trauma, principalmente para lidar com membros isquêmicos, controlar hemorragias e ajudar em exposições cirúrgicas complexas, vem sendo cada vez mais requisitada^11,14,33^ . Entretanto, treinamento inadequado em trauma vascular pode impactar negativamente na evolução dos casos. Cerca de 70% das lesões vasculares traumáticas acometem os membros inferiores^3,25^ , sendo a artéria femoral superficial o vaso mais frequentemente atingido^1,34^ . As lesões de vasos poplíteos são responsáveis por altos índices de amputação^5,18,19,23,24^ , ressaltando a importância do estudo sobre fatores prognósticos associados a esses ferimentos.

Os vasos femorais profundos raramente são lesionados, e sabe-se que sua ligadura não representa uma questão crítica nem para a perfusão arterial e nem para drenagem venosa do membro^18^ . Por essas razões, lesões desses vasos não foram incluídas neste estudo. Pacientes com lesões de outras topografias, que pudessem confundir a causa do óbito, também foram excluídos da análise quanto a este desfecho. Todas as análises foram feitas com o objetivo de avaliar apenas a repercussão das lesões vasculares estudadas sobre o prognóstico dos pacientes. Foram consideradas reintervenções cirúrgicas apenas aquelas relacionadas às lesões vasculares. Embora todos os pacientes estejam sujeitos a lesões vasculares traumáticas, a vasta maioria acomete homens jovens^1-5,7,11,12,33^ , como detectado em nosso estudo.

A etiologia do trauma vascular não é uniforme. Nos continentes americano e africano, as lesões por arma de fogo e armas brancas são mais frequentes^2,3^ , em consonância com esta casuística. Os vasos mais acometidos foram os femorais superficiais, corroborando a literatura^1,8,17,33,34^ . Lesões da artéria femoral superficial levam à perda do membro em 7 a 13% dos casos^3,35^ , concordando com nosso estudo, em que 8,3% dos indivíduos com esta lesão evoluíram para amputação.

A artéria poplítea foi a segunda mais lesionada nesta casuística; estas lesões podem alcançar taxas de amputação de 26%^5^ . Nesta casuística, os sete casos de lesão de artéria poplítea evoluíram para amputação, o que se justifica pelo pequeno número de casos e pelas grandes distâncias percorridas pelos pacientes até o atendimento. Quanto ao tratamento dos traumatismos arteriais, o enxerto com veia autóloga, descrito na literatura como a técnica mais comum para correção dessas lesões^18,23,27^ , foi utilizado em 59,6% dos casos. Quando a ressecção dos cotos arteriais danificados permite uma anastomose término-terminal sem tensão, essa pode ser a técnica escolhida^2,21,25^ - e nesta amostra, foi a segunda estratégia mais aplicada (23,6% dos casos). Para o tratamento das lesões venosas, são descritas venorrafia, anastomose término-terminal, interposição de enxerto e ligadura^12,33^ . Nesta amostra, a ligadura foi aplicada em 81,4% dos casos, seguida pela venorrafia, em 13,6% dos pacientes.

Lesões vasculares das extremidades podem estar associadas a traumatismos esqueléticos ou de outras topografias^1,2,5,18,25,27^ , sendo que lesões associadas acometeram 69,2% dos casos desta amostra. Nos traumatismos contusos, lesões associadas são mais frequentes e potencializam o risco de amputação^1,5,18,25-27^ . Os resultados deste estudo sustentam esse raciocínio, pois foi detectada associação estatisticamente significativa entre traumatismo contuso e amputação do membro. Também é mais comum que esses resultem em síndrome compartimental, pela associação entre fraturas e lesões vasculares^1,5,18,21,26,27^ , e, de fato, nossos resultados demonstraram que a necessidade de fasciotomias esteve associada a maior frequência de amputação.

Vários trabalhos já comprovaram que o transporte rápido, agilizando o atendimento hospitalar, é determinante para a melhor evolução dos casos^3,14-16,18,19^ , e que o choque hipovolêmico e a reperfusão precoce do membro traumatizado são os pilares do tratamento nos traumatismos vasculares das extremidades^1,14,18^ . Esta pesquisa reflete isso, pois a quantidade significativa de pacientes que já se apresentaram em choque na admissão (37,2%) está ligada ao fato do hospital onde a pesquisa foi realizada atender uma área de 1.248.000 km^2^, onde o resgate aeromédico frequentemente está indisponível^1,36^ . Assim, aproximadamente 50% dos pacientes precisaram ser transportados por mais de 100 km por via terrestre e fluvial até o atendimento, impactando negativamente nos desfechos clínicos da população estudada.

Um dos fatores prognósticos mais polêmicos discutidos na literatura é o fato da ligadura venosa possivelmente predispor à necessidade de amputação do membro. Muitos trabalhos já afirmaram essa correlação^14,29^ ; entretanto, diversos autores contemporâneos sugerem que, na realidade, a ligadura venosa não aumenta esse risco, e que o reparo das veias traumatizadas pode aumentar o risco de tromboembolismo^28,29^ . Nossos resultados acompanham essas constatações, já que não foi demonstrada associação estatisticamente significativa entre ligadura venosa e evolução para amputação nas lesões das três topografias estudadas.

Não foram localizadas pesquisas anteriores que tenham avaliado o impacto de decisões cirúrgicas inapropriadas sobre a evolução das vítimas de lesões vasculares. Este estudo permitiu evidenciar situações em que a técnica empregada contrariou princípios clássicos. Houve casos de ferimentos por arma de fogo em que a lesão arterial foi tratada apenas com trombectomia seguida de arteriorrafia, sem ressecção do segmento traumatizado. Essa estratégia mantém o endotélio traumatizado, predispondo à trombose e consequente isquemia^25,28,37,38^ . Também foram detectados casos em que, havendo lesão arterial e venosa simultâneas, a veia foi tratada por ligadura, e o cirurgião optou pela retirada da safena magna do membro traumatizado para reconstrução arterial. No entanto, a recomendação clássica é a utilização da safena contralateral, pois a diminuição da drenagem pelo sistema venoso superficial prejudica a compensação do retorno venoso do membro submetido à ligadura de veias profundas, acarretando congestão venosa, síndrome compartimental e isquemia irreversível^18,25,37,39^ .

A gravidade do quadro não justifica a realização destas manobras, classicamente descritas na literatura como inadequadas^37-39^ . Se, por instabilidade hemodinâmica, ausência de materiais necessários ou por outras razões, não for possível o uso de técnicas corretas, recomendam-se estratégias de “controle de danos”, capazes de impactar positivamente no prognóstico do membro traumatizado^2,3,10,17,25^ . O fato de 37,2% dos pacientes terem sido admitidos em choque hipovolêmico contrasta com a descrição esporádica de técnicas de controle de danos nessa amostra, como o *shunt* vascular temporário, ferramenta cujo domínio é obrigatório para cirurgiões que lidam com esses traumatismos^2,3,9,11,17,25^ .

Todos os casos analisados neste estudo foram operados por especialistas em cirurgia vascular. Apesar disso, conforme demonstrado, a não observância de conceitos tradicionais na condução de traumatismos vasculares foi relativamente frequente e esteve associada a desfechos desfavoráveis. A justificativa para essas ocorrências provavelmente é multifatorial: a heterogeneidade dos membros da equipe quanto ao treinamento em trauma vascular durante a residência médica e à experiência individual acumulada podem ser alguns desses fatores, ressaltando a necessidade de aperfeiçoamento específico quanto ao tratamento de lesões vasculares traumáticas, com ênfase em estratégias de controle de danos.

A literatura descreve pontuações elevadas no ISS como fator prognóstico importante quanto à sobrevida das vítimas de trauma vascular^6,18,22,25^ . Entretanto não foi detectada diferença estatisticamente significativa entre o ISS das vítimas fatais e dos pacientes que sobreviveram, provavelmente devido ao tamanho da amostra, que também explica porque a ocorrência de choque na admissão hospitalar, descrita como fator ligado a maior mortalidade^2,6,31,32^ , não ter sido associada de forma estatisticamente significativa ao óbito. Embora tenhamos avaliado todos os casos que preenchiam os critérios para inclusão atendidos durante o período estudado, o cálculo de tamanho amostral de uma população finita foi posteriormente realizado, sendo que para essa população uma amostra de 76 pacientes seria considerada significante (Figura 4). Trauma é um campo de estudo peculiar. Os casos têm mecanismos e evoluções heterogêneas, e frequentemente a gravidade dos quadros clínicos dificulta o adequado registro de uma série de variáveis.

**Figura 4:** Cálculo do tamanho amostral.

Entre as limitações deste estudo, destaca-se o caráter retrospectivo da pesquisa, com preenchimento incompleto de prontuários e descrições cirúrgicas nem sempre precisas. Além disso, apesar do número de casos ter superado o tamanho mínimo calculado para uma amostra significativa, é possível que a realização de projetos prospectivos multicêntricos, com uma amostra maior, diminua essas limitações. Os autores sugerem a avaliação de decisões cirúrgicas inadequadas entre as variáveis pesquisadas, já que esta pesquisa preliminar sugere que este seja um importante fator associado ao prognóstico desfavorável destes pacientes.

## CONCLUSÃO

A maioria das lesões vasculares do segmento femoropoplíteo acometeu homens de aproximadamente 30 anos, vítimas de ferimento por projéteis de arma de fogo. Lesões de vasos femorais superficiais foram mais frequentes que as de vasos poplíteos, e os vasos femorais comuns foram os menos afetados. A associação com lesões concomitantes de estruturas não vasculares foi comum, sendo as fraturas as mais frequentes. O enxerto venoso foi o tratamento mais utilizado no trauma arterial, e a ligadura foi o mais empregado para lesões venosas. Traumatismos contusos, provocados por acidente de trânsito, estiveram mais associados à amputação do membro, quando comparados aos demais mecanismos de trauma. A ligadura venosa não aumentou a probabilidade de amputação dos membros traumatizados. Decisões cirúrgicas inadequadas resultaram em maior probabilidade de reintervenções. Lesão de artéria poplítea e necessidade de realização de fasciotomia aumentaram a probabilidade do membro traumatizado evoluir para amputação. Nenhuma das variáveis pesquisadas apresentou correlação estatisticamente significativa para evolução fatal.
